# UNDERSTANDING THE NEW NIH DATA SHARING REQUIREMENTS: WHY THEY IMPROVE THE QUALITY OF RESEARCH

**DOI:** 10.1093/geroni/igad104.1532

**Published:** 2023-12-21

**Authors:** James McNally

**Affiliations:** University of Michigan, Ann Arbor, Michigan, United States

## Abstract

In 2021, NIH began a long period of planning, drafting, and requesting public comment to create a new data-sharing policy, beginning with a Request For Information (RFI). The new policy, beginning January 25, 2023, will apply broadly to all scientific data produced with NIH funding. NACDA and (ICPSR), drawing upon our expertise and understanding of best practices for data sharing, have been working actively to develop tools and resources to assist NIH grant recipients in complying with federal data-sharing expectations with minimal disruptions to their research. This presentation will provide a detailed overview of the new data-sharing requirements and how to incorporate them into the required data-sharing plan. We will offer examples of successful data-sharing plans, identifying an appropriate repository, budgeting for the data preparation process, and creating informed consent documents consistent with the spirit of the NIH Data Sharing requirements. In addition to explaining the new data-sharing policy, the presentation will discuss the rationale behind the requirements and how they contribute to data equity using the FAIR principles. By increasing access to federally funded data, we contribute to a multidisciplinary approach that enhances cross-disciplinary communication and a broad understanding of different ways of knowing when working collaboratively with other researchers. Team Science has facilitated substantive discourse between qualitative and quantitative specialists. Multidisciplinary research increasingly represents a critical aspect of overall knowledge creation. Finally, the presentation will illustrate how data-sharing increases the impact of studies and enhances the credentials of researchers who engage in data-sharing.

